# Conflict in the Indian Kashmir Valley II: psychosocial impact

**DOI:** 10.1186/1752-1505-2-11

**Published:** 2008-10-14

**Authors:** Kaz de Jong, Saskia van de Kam, Nathan Ford, Kamalini Lokuge, Silke Fromm, Renate van Galen, Brigg Reilley, Rolf Kleber

**Affiliations:** 1 Médecins Sans Frontières, Plantage Middenlaan 14, 1018 DD Amsterdam, the Netherlands; 2 Faculty of Health Sciences, Simon Fraser University, Vancouver, Canada; 3 Department of Clinical Psychology, Utrecht University, the Netherlands

## Abstract

**Background:**

India and Pakistan have disputed ownership of the Kashmir Valley region for many years, resulting in high levels of exposure to violence among the civilian population of Kashmir (India). A survey was done as part of routine programme evaluation to assess confrontation with violence and its consequences on mental health, health service usage, and socio-economic functioning.

**Methods:**

We undertook a two-stage cluster household survey in two districts of Kashmir (India) using questionnaires adapted from other conflict areas. Analysis was stratified for gender.

**Results:**

Over one-third of respondents (n = 510) were found to have symptoms of psychological distress (33.3%, CI: 28.3–38.4); women scoring significantly higher (OR 2.5; CI: 1.7–3.6). A third of respondents had contemplated suicide (33.3%, CI: 28.3–38.4). Feelings of insecurity were associated with higher levels of psychological distress for both genders (males: OR 2.4, CI: 1.3–4.4; females: OR 1.9, CI: 1.1–3.3). Among males, violation of modesty, (OR 3.3, CI: 1.6–6.8), forced displacement, (OR 3.5, CI: 1.7–7.1), and physical disability resulting from violence (OR 2.7, CI: 1.2–5.9) were associated with greater levels of psychological distress; for women, risk factors for psychological distress included dependency on others for daily living (OR 2.4, CI: 1.3–4.8), the witnessing of killing (OR 1.9, CI: 1.1–3.4), and torture (OR 2.1, CI: 1.2–3.7). Self-rated poor health (male: OR 4.4, CI: 2.4–8.1; female: OR 3.4, CI: 2.0–5.8) and being unable to work (male: OR 6.7, CI: 3.5–13.0; female: OR 2.6, CI: 1.5–4.4) were associated with mental distress.

**Conclusion:**

The ongoing conflict exacts a huge toll on the communities' mental well-being. We found high levels of psychological distress that impacts on daily life and places a burden on the health system. Ongoing feelings of personal vulnerability (not feeling safe) was associated with high levels of psychological distress. Community mental health programmes should be considered as a way reduce the pressure on the health system and improve socio-economic functioning of those suffering from mental health problems.

## Background

The Partition of India in 1947 was the start of a long history of dispute between India and Pakistan for control of Kashmir, which today remains divided into three parts governed by India, Pakistan and China. Over the last 20 years, a liberation struggle between India and Kashmiri militants has led to at least 20,000 deaths and 4,000 disappearances in the Indian part of Kashmir [1].

A community survey done by Médecins Sans Frontières in 2005 found high levels of ongoing violence across the region, with civilians caught in the middle. The majority of people surveyed stated having been exposed to crossfire (86%) and round-up raids (83%). High numbers of people reported being subjected to maltreatment (44%), forced labour (33%), kidnapping (17%), torture (13%) and sexual violence (12%). [2]

Exposure to violence has potentially important implications for mental health [3]. This paper presents the findings of the community assessment survey done by Médecins Sans Frontières in 2005. The study, which was done to inform program planning, assessed the mental health and socio-economic impact of the ongoing violence, and the sources of support.

## Methods

The survey was conducted in mid-2005 in the Indian part of Kashmir (Kupwara and Badgam, totalling 101 villages and a combined population 145,000 people). The methodology is described in detail elsewhere [2]. Briefly, sample size calculation assumed a prevalence of trauma-related psychological problems of 20% [4]; using a precision of 5% (confidence interval 95%) and a design effect of 2, the minimum sample size was estimated at 492. A two-stage cluster sampling design was used to cover 30 villages (randomly selected), resulted in 17 households per village. Within the household participants aged ≥ 18 years were selected randomly. Informed consent was attained for all participants and MSF's independent Ethical Review Board granted ethical approval.

### Instruments

The overall survey questionnaire assessed baseline demographics, confrontation with violence (results presented elsewhere [2]), mental health, health service usage, socio-economic functioning and sources of support. Mental health was assessed using a Self-Reporting Questionnaire (SRQ), with a reference period of 30 days preceding the survey. The SRQ is an instrument developed by the World Health Organization (WHO) to measure general psychological distress, especially in developing countries. It has good validity and reliability for adults (≥ 15 years) [5], and can be used both as a self- or interviewer-administrated questionnaire. It consists of 20 closed questions covering expression of distress, the total score corresponding to the sum of positive responses. Various studies have validated the use of the SRQ in India [6-9]. Currently a cut off score of 11 or 12 is accepted [10] although this has been critiqued as being too high [11]. In our study we used a conservative cut-off score of 12, meaning those respondents scoring ≥ 12 are considered to be suffering from psychological distress.

Four categories of closed questions were applied to establish use of health services (categories: never; once; 2–3 times; 4+) and medications (Categories: never; 1–3 times; 4–6 times; 7+). Closed questions were also used to assess coping mechanisms for dealing with stress. The composition of categories for 'consequences of violence' and 'sources of support' was done with input from national staff.

To establish individual socio-economic functioning in relation to health during the past thirty days the H-section of the WHO-Disability Assessment Schedule-II (WHO-DAS-II) was used. This tool has good internal, convergent validity and good sensitivity for change [12].

The survey was forwarded and back translated from English to Urdu and phonetic Kashmiri and piloted prior to full implementation.

### Analysis

Data entry was standardised and checked by supervisors, entered into EXCEL and analysed in EPIINFO-2002. Because males and females differed significantly in the number of confrontations with violence [2], we used univariate analysis to stratify for gender to determine relationships between psychological distress (SRQ ≥ 12) and demographic details, living circumstances, confrontations with violence (witnessing, self-experiencing), health outcomes (physical symptoms, health service use), and socio-economic functioning. We excluded variables such as 'torture while being detained/held hostage' as these responses relate to a sub-sample of those surveyed. We also excluded exposure to violence from this analysis because the proximity to the violence was not defined in detail.

A multivariate statistical model was constructed to investigate relationships between mental health (SRQ ≥ 12) and the above-mentioned variables. We used a logistic regression model including variables that were significant in the univariate analysis (p < 0.05) with backward elimination. In our model we expected each type of event conferring an additional risk over and above any other event experienced. This is in accordance with studies reporting exposure to cumulative traumatic events as a risk factor for the development of PTSD [13,14].

## Results

510 of 548 (93%) interviews were completed. Reasons for refusal to participate (25) and stopping the interview (13) included: lack of time, distrust, and being emotionally upset. The average age of respondents was 37.7 years (range 17–90) with an equal gender distribution (males = 53%; 270; p > 0.05). Demographics are described in detail elsewhere. [2]

### Mental health status

Psychological distress was mostly expressed through symptoms such as nervousness, tiredness, being easily frightened and headache (Table 1). The prevalence of suicidal ideation is striking: one-third of those surveyed had had thoughts of ending their life in the past 30 days. Over a third of respondents were categorized as suffering from psychological distress (SRQ ≥ 12) using the Indian validated SRQ (33.3%, 170; CI: 28.3–38.4). The design effect for the SRQ was 1.4. Females scored significantly higher (43.8% vs. 24.1%, OR 2.5; CI: 1.7–3.6; p < 0.001).

**Table 1:** Self-reporting questionnaire 20 (n = 510)

	**Items SRQ 20**	**YES**		**Items SRQ 20**	**YES**
1	Do you often have headaches?	53.6% (272)	11	Do you find it difficult to enjoy your daily activities?	50.0% (255)
2	Is your appetite poor?	40.8% (208)	12	Do you find it difficult to make a decision?	39.6% (202)
3	Do you have sleep disturbances?	45.5% (232)	13	Is your daily work suffering?	51.8% (264)
4	Are you easily frightened?	55.9% (285)	14	Do you feel you are usefully contributing in life?*	31.0% (158)
5	Do you feel nervous, tense, or worried?	62.7% (320)	15	Have you lost interest in things?	45.1% (230)
6	Do your hands tremble?	50.2% (256)	16	Do you feel that you are a worthless person?	37.8% (193)
7	Is your digestion poor?	25.1% (128)	17	Have you thought about ending your life?	33.9% (173)
8	Do you have trouble thinking clearly?	50.2% (256)	18	Do you feel tired all the time?	62.5% (319)
9	Do you feel unhappy?	50.0% (255)	19	Do you have uncomfortable feelings in your stomach?	39.8% (203)
10	Do you cry more than usual?	45.1% (230)	20	Are you easily tired?	66.7% (340)

• This question was changed from the original SRQ 20 questionnaire (Are you unable to play a useful part in life?).

• In the current format the No-answer was used as sign of psychological distress).

### Associations between psychological distress (SRQ ≥ 12) and violence, health, socio-economic and sources of support

#### Univariate analysis of violence and psychological distress (SRQ ≥ 12)

Feelings of personal insecurity were significantly associated with psychological distress (SRQ ≥ 12) for both males and females (Table 2). Psychological distress among males was significantly (p < 0.01) associated with all self-experiences (defined as 'ever happened to you') and most consequences of violence. Psychological distress among females was significantly (p < 0.01) associated with witnessing events (except hearing about/witnessing rape), as well as the self-experience of some events (maltreatment, arrested/kidnapped) and feelings of lack of safety and independence.

**Table 2:** Univariate analysis of cases (SRQ ≥ 12) with non-cases on demographic variables, living circumstances, confrontations with violence (self-experience, witnessing), and personal consequences stratified by gender (n = 510)

**Variable**	**SRQ ≥ 12 Males n = 270**				**SRQ ≥ 12 Females n = 240**				**SRQ ≥ 12 all n = 510**			
	**N**	**OR**	**CI**	**P**^i^	**N**	**OR**	**CI**	**P**^i^	**N**	**OR**^iii^	**CI**	**P**^iiii^
**Demographics**												
Marital status												
-*Not married*	65	1			60	1			125	1		
-*Married*	203	1.7	0.8–3.6	0.178	176	1.8	1.0–3.3	0.865	379	1.8*	1.1–2.8	0.023
**Living**												
**Circumstances**												
Currently Feeling Safe												
-*Always/most*	144	1			120	1			264	1		
-*Occasionally/never*	126	2.3 **	1.3–4.1	0.006	118	2.0 *	1.2–3.3	0.014	224	2.1**	1.4–3.1	0.000
Dependency for Living												
-*Self supportive, nearly*	194	1			185	1			379	1		
-*Highly, total dependant*	74	1.6	0.9–3.0	0.147	53	2.4 **	1.3–4.6	0.007	127	2.0**	1.3–3.1	0.002
Having Two meals a day												
-*Always, sometimes*	258	1	1.1–15.9		229				487	1		
-*Rarely, never*	9	4.1		0.068	7	1.8	0.4–8.1	0.352 ^ii^	16	2.8	1.0–7.6	0.07
**Witnessing**												
Seeing wounded people												
-*No*	73	1			115	1			188	1		
-*Yes*	197	2.1*	1.1–4.5	0.043	125	1.8*	1.0–3.1	0.030	322	2.0**	1.3–3.0	0.002
Witnessed people being arrested												
-*No*	44	1			81	1			125	1		
-*Yes*	226	2.8*	1.1–7.7	0.044	159	2.0*	1.2–3.6	0.018	385	2.3**	1.4–3.7	0.001
Witnessed people being killed												
-*No*	151	1			155	1			306	1		
-*Yes*	119	1.6	0.9–2.8	0.123	85	2.0*	1.6–3.4	0.018	204	1.8**	1.2–2.6	0.004
Witness people being tortured												
-*No*	68	1			101				169	1		
-*Yes*	202	1.7	0.9–3.4	0.179	139	2.3**	1.4–4.0	0.003	341	2.1**	1.3–3.1	0.001
Witnessed people being maltreated/molested												
-*No*	46	1			90				136	1		
-*Yes*	224	1.6	0.7–3.8	0.301	150	2.1*	1.2–3.6	0.016	374	1.9**	1.2–3.1	0.005
Heard about cases of rape												
-*No*	67	1			117	1			184	1		
-*Yes*	203	2.2	1.0–4.5	0.054	123	1.0	0.6–1.6	0.958	326	1.3	0.9–2.0	0.256
Witnessed rape												
-*No*	223	1			219	1			442	1		
-*Yes*	47	1.8	0.9–3.5	0.134	21	1.8	0.7–4.5	0.269	68	1.8*	1.0–3.1	0.045
**Self-experienced**												
Being maltreated												
-*No*	110	1			175	1			285	1		
-*Yes*	160	2.4**	1.3–4.5	0.007	65	2.0*	1.1–3.5	0.032	225	2.2**	1.4–3.3	<0.001
Being forced to do labour												
-*No*	144	1			194	1			338	1		
-*Yes*	126	2.5**	1.4–4.4	0.002	46	1.4	0.7–2.7	0.396	172	1.9**	1.3–2.9	0.003
Being forced housing any of the parties												
-*No*	203	1			213	1			426	1		
-*Yes*	67	2.4**	1.3–4.3	0.008	27	0.7	0.3–1.7	0.621	94	1.53^x^	0.95–2.5	0.100
Being arrested/kidnapped									424			
-*No*	195				229	1			86	1		
-*Yes*	75	3.2***	1.8–5.8	< 0.001	11	6.3**	1.3-30.0		0.010^ii^		3.6**	2.1–6.2	<0.000
Modesty being violated												
-*No*	224	1			227	1			451	1		
-*Yes*	46	4.2***	2.1–8.1	<0.000	13	2.2	0.7–6.9	0.1416	59	3.5**	2.0–6.2	<0.000
Being injured because of conflict												
-*Not injured*	248	1			234	1			484	1		
-*Injured*	22	4.3 **	1.8–10.5	0.002	6	2.7	0.5-14.9	0.452	28	3.8**	1.7–8.5	0.001
**Consequences of violence**												
Moving voluntarily for safety reasons												
-*No*	131	1			143	1			274	1		
-*Yes*	139	2.3**	1.3–4.1	0.007	97	1.8*	1.0–3.0	0.048	236	2.0**	1.3–2.9	<0.000
Forced to move (being displaced)												
*No*	221	1			199	1			420	1		
*Yes*	48	4.2***	2.2–8.2	<0.000	40	2.0	1.0–3.9	0.075	88	2.9**	1.8–4.6	<0.000
Being disabled												
-*No*	232	1			228	1			460	1		
-*Yes*	38	3.9***	1.9–8.0	<0.000	10	3.2	0.8-12.7	0.079	48	3.7**	2.0–7.1	<0.000
Having lost house												
-*No*	253	1			225	1			478			
-*Yes*	17	1.3	0.4–3.9	0.404	13	1.6	0.5–4.9	0.592	30	1.5	0.7–3.1	0.468
Having lost possessions												
-*No*	197	1			183	1			380	1		
*Yes*	73	2.6**	1.4–4.5	0.002	57	1.6	0.9–3.0	0.1417	130	2.1	1.3–3.1	0.001

i P Chi square Yates corrected unless indicated differently

ii Fisher exact test

iii OR adjusted for gender

iiii P Mantel Heinzel Chi square corrected unless indicated differently

* Signifiant P < 0.05

** Significant P < 0.01

*** Significant P < 0.001

χ Chi-square for differing Odds Ratios by gender is significant (p = 0.028) suggesting interaction

#### Multivariate analysis of mental health (SRQ ≥ 12) and violence

For both genders, not feeling safe is associated with at least twice the odds of suffering from psychological distress (Table 3). For males, violation of modesty, forced displacement, and disability were all associated with a significantly increased likelihood (three times the odds) of suffering from psychological distress. For women, the witnessing of people being killed or tortured or dependency on outside assistance doubled the odds of suffering psychological distress.

**Table 3:** Significant multivariate associations between psychological distress (SRQ ≥ 12) and demographic variables, violent incidents (self-experience, witnessing), and personal consequences by gender (*n *= 510)

	**OR**	CI	P-value
**MALE SRQ ≥ 12**
Currently not feeling safe			
*No*	1		
*Yes*	2.4**	1.3–4.4	0.007
Modesty being violated			
*No*	1		
*Yes*	3.3**	1.6–6.8	0.001
Being forced to move			
*No*	1		
	3.5***	1.7–7.1	<0.001
Being disabled			
*No*	1		
*Yes*	2.7*	1.2–5.9	0.015
**FEMALE **SRQ ≥ 12			
Currently not feeling safe			
*No*	1		
*Yes*	1.9*	1.1–3.3	0.020
Being dependent for daily living			
*No*	1		
*Yes*	2.4**	1.3–4.8	0.007
Witnessed people being killed			
*No*	1		
*Yes*	1.9*	1.1–3.4	0.029
Witnessed people being tortured			
*No*	1		
*Yes*	2.1**	1.2–3.7	0.008

^i ^Multi logistic regression

* Signifiant P < 0.05

** Significant P < 0.01

*** Significant P < 0.001

#### Associations between psychological distress (SRQ ≥ 12), health and socio economic outcomes

The majority of respondents (63.9%, 326) had recently visited a health postor clinic: nearly half had visited a health facility more than once (46.3%, 235) in the past 30 days. Overall, nearly half (49.6%, 253) of respondents rated the health facilities as poor. Women more frequently rated their physical health as bad or very bad (male: 24.1% vs. female: 36.3%, OR 1.8; CI: 1.2–2.6; p < 0.005), and visited the health facilities more than men (male: 40.0% vs. female: 54.7%, OR 1.8; CI: 1.3–2.6; p = 0.005). The number of women who had been on medication for six or more days was significantly higher than men (male: 30.7% vs. female: 46.0%, OR 1.9; CI: 1.3–2.8; p < 0.001). A high level of psychological distress (SRQ ≥ 12) was significantly (p < 0.01) associated with poor or very poor self-rated health for both males (OR 4.4) and females (OR 3.4). For males this was also associated with a higher likelihood of visiting the clinic two times or more (Table 4). For both males and females, high psychological distress was also associated with a higher likelihood of being unable to or having to cut back on work or performance of daily activities.

**Table 4:** Associations between psychological distress (SRQ >= 12) and health outcomes, socio-economic outcomes by gender (n = 510)

	**Males**				**Females**			
	**n**	**OR**	**CI**	**P value**	**n**	**OR**	**CI**	**P value**
**Health Outcomes**
Self rated health bad or very bad								
*SRQ < 12*		1				1		
*SRQ ≥ 12*	65	4.4**	2.4–8.1	<0.0001	87	3.4**	2.0–5.8	<0.0001
Visited health clinics ≥ 2 times								
*SRQ < 12*		1				1		
*SRQ ≥ 12*	106	3.2**	1.8–5.8	<0.0001	129	1.4	0.9–2.4	0.166
Medicine use > 6 days								
*SRQ < 12*		1				1		
*SRQ ≥ 12*	81	1.8	1.0–3.1	0.006	106	1.5	0.9–2.6	0.11
**Socio-economic Outcomes**								
Unable to work/daily activities ≥ 4 days								
*SRQ < 12*		1				1		
*SRQ ≥ 12*	117	6.7**	3.5–13.0	<0.0001	124	2.6**	1.5–4.4	<0.001
Cut back/reduce work or daily activities ≥ 4 days								
*SRQ < 12*		1				1		
*SRQ ≥ 12*	120	4.1**	2.2–7.6	<0.0001	125	4.5**	2.6–8.0	<0.001

i P Chi square Yates corrected unless indicated differently

ii Fisher exact test

* Significant P < 0.05

** Significant P < 0.01

*** Significant P < 0.001

#### Coping mechanisms

The most common ways of coping were withdrawal (isolation, not talking to people) and aggression (Table 5). Religion was also reported as a helpful source of support.

**Table 5:** Overview support mechanism used by the participants (up to three answers possible, n = 510).

**Sources of support**	**Frequency**
Isolation	327 (64.1%)
Aggressive behaviour	235 (46.1%)
Praying/meditation	203 (39.8%)
Stop speaking to people	188 (36.9%)
Drug and alcohol use	186 (36.5%)
Talking to others	117 (22.9%)
Keeping busy	106 (20.8%)
Seeking support from family	63 (12.4%)
Other	44 (8.6%)

## Discussion

The data presented in this article were gathered to inform MSF's programme to provide mental health support in Kashmir. Using the SRQ (a tool that has been validated in other Indian studies [6-10]) we found the population had been exposed to high levels of violence [2] which resulted in one third of the respondents suffering from psychological distress and considering suicide. For both genders, currently not feeling safe was associated with psychological distress. For males 'violation of modesty', displacement, and disability were associated with psychological distress while risk factors for females included witnessing killing and torture. Respondents with high psychological distress rated their own health and socio economic functioning as poor. The most common coping mechanism was withdrawal.

Overall, one-third of respondents reported psychological distress. This compares to a prevalence of 36% found in a study done in among Afghan women in a refugee camp [15] using the same instrument and similar cutoff score, but differs substantially from another SRQ study done in a non-conflict area in India [16] where 18% prevalence of psychological distress was found among low-income urban women, using a relatively low cut-off score (7/8). (Using this lower cut-off would have given a prevalence of psychological distress of 71.4%). The contextual difference in these studies – exposure to chronic violence as compared to 'common' stressors of daily life for women in low urban settings – may account for this difference.

The Self Reporting Questionnaire (SRQ) showed that a third of respondents had contemplated suicide. Suicidal thoughts are common for depressive disorders [17] but do not always lead to a suicide attempt. Our findings are in line with a previous study that reported high suicide rates in this region [18]. A high prevalence of suicidal thoughts is more often reported among populations suffering from chronic violence, with a similar prevalence (33%, 96, n = 297) reported in a population of Afghan refugee women in Pakistan using the same questionnaire (SRQ).

In our study women had significantly higher psychological distress than man. This is in line with other studies showing women suffering more from anxiety disorders than men after confrontation with violence [20]. Feeling safe was found in other studies to be an important pre-condition for being able to deal with adverse traumatic experiences [21,22], and this was also found in our study.

For males, the most important risk factors for developing psychological distress were 'violation of modesty', displacement and disability. It is possible that these experiences are the most distressing because they interfere with the cultural values and roles of males in Kashmir society: upholding their dignity and being able to protect and feed their families. Those who self-experienced 'violation of modesty' had a threefold chance of suffering from psychological distress (p = 0.001). 'Violation of modesty' is regarded as very degrading and in the few studies on male sexual violence is associated with multiple perpetrators and high levels of physical beating [23,24], which can further contribute to psychological distress.

For women most psychological distress was associated with feelings of powerlessness – dependency on others for daily living, and witnessing killing and torture. Women have lower confrontations with violence, which can be partly explained by their being largely confined to the home [2]. The significant association of witnessing and psychological distress among females may relate to feelings of helplessness and guilt caused by the witnessing may be more traumatic than experiencing the violence themselves.

Both males and females with high levels of psychological distress rated their own health as much poorer compared to those who did not have high levels of psychological distress (male: OR 4.4; female: OR 3.4). Non-specific health complaints have been associated with (traumatic) stress in other studies [25-27]. It is also possible that people do not understand the relationship between physical symptoms and mental stress [28] or have difficulty to articulate their emotional status and use physical symptoms to articulate mental distress [29].

High psychological distress among males was significantly associated with visiting health services more frequently. Increased use of medical services by those suffering from traumatic-stress related problems are common [30,31], with up to a 25% increase in number of visits to health care facilities reported in other studies [32-34]. We found this relationship in our survey for males, but not for females. This may be explained by the fact that for both cultural and security reasons females depend on male escorts in order to access health services, restricting their movements.

In our population, high psychological distress is associated with substantially increased likelihood of socio-economic dysfunction, and this has been reported in both Western [35,36] and Asian [15] contexts. Socio-economic dysfunction can have broad implications, for example by reducing capacity of females to give care to the children or for males to generate income (according to traditional roles).

The most common coping mechanisms such as withdrawal (self-isolation, stop speaking) and aggression may also be symptomatic of depression and/or anxiety disorder (including post-traumatic stress disorder, PTSD). Religion and family assistance are mentioned less frequently as sources of support. This is in contrast to a study conducted in Afghanistan that showed religion and reading the Koran as the two main coping mechanisms for two being confronted with violence [15].

### Potential limitations

General methodological limitations, including sampling methodology, retrospective study design, and terminology, have been discussed previously [2]. There are, in addition, a number of potential limitations related to this specific analysis. First, as this is a cross-sectional survey, no causal inferences between violence and mental health can be conclusively made. Second, individual respondents may have implicitly used the presence of mental health symptoms as a deciding factor for whether they have experienced a traumatic event in case of doubt (i.e. recall bias [37]). We consider this as unlikely as we asked respondents to recall violent events but did not ask them to identify which events were traumatic. Finally, we used the SRQ to avoid labelling populations with a psychiatric diagnosis, but using a self-reporting questionnaire has obvious limitations. A comparative study in India of five questionnaires showed good internal consistency and a high discriminating ability with the SRQ having the best results [9], but in comparison to clinical interview, questionnaires only showed strong positive predictive value when a considerable compromise on sensitivity was made. It was concluded that the choice of an optimum cut-off score (to balance sensitivity and positive predictive value) should be adapted to individual settings, and recommend a higher cut-off score for resource-limited primary-care settings [9]. We used a high cut off score of 12, in line with this recommendation. But in the absence of clinical interview no detailed analysis of the mental health status is possible.

In the context of predominantly Urdu speaking population we considered, but did not use, cut off scores from other Urdu speaking cultures such as in Pakistan. A meta-analysis of psychiatric rating scales in Urdu [38] concluded that only a small number of instruments (including SRQ) were sufficiently evaluated. The same review concluded that for the SRQ no cross-culturally validated gold standard was used, cut-offs varied considerably, as did sensitivity (78–93%) and specificity (77–85%). We consider the Indian validation studies [9] as more appropriate because they used clinical interview as gold standard.

## Conclusion

The high levels of violence confronted by the Kashmiri population have resulted in high prevalence (33%) of mental health problems. Poor self-rated health and likelihood of poor socio-economic functioning were associated with high levels of psychological distress. Mental health problems in this context of chronic violence should receive full attention through the provision of appropriate community-based services that would improve access to care and reduce the burden on the health system.

## Conflicts of interests

The authors declare that they have no competing interests.

## Authors' contributions

KJ designed and co-ordinated the study and wrote the first draft of the paper. NF supported the conceptual framing of the findings, assisted with the analysis, and led subsequent drafts. SK and KL provided statistical support for the design and analysis, and helped with the writing of the paper. SF, RG and BR oversaw the implementation of the survey, managed data collection in the field, and contributed to the writing of the paper. RK provided conceptual oversight and contributed to the writing of the paper.
